# Acceptability of Sharing Internet Browsing History for Cancer Research: Think-Aloud and Interview Study

**DOI:** 10.2196/82009

**Published:** 2026-02-02

**Authors:** Nicola Cara Gradwell, Mel Ramasawmy, Sanjula Arora, Manoj Mistry, Reshma Punjabi, Christina Derksen, Suzanne E Scott

**Affiliations:** 1 Wolfson Institute of Population Health, Queen Mary University of London, Charterhouse Square, London, EC1M 6BQ, United Kingdom, 44 020 7882 3850

**Keywords:** cancer, digital inequalities, early diagnosis, health inequalities, health-seeking behavior, internet search, internet search data, smart data, web search

## Abstract

**Background:**

Growing interest surrounds how internet search behaviors might provide digital signals of disease prior to diagnosis, for example, when people search symptoms online. Internet browsing data offer novel opportunities for understanding response to symptoms, public health surveillance, and early intervention in conditions such as cancer. However, the acceptability of using such sensitive data in medical research remains unclear, particularly among individuals at higher risk of health and digital exclusion, such as older adults and those from minority ethnic groups or with a lower socioeconomic status.

**Objective:**

This study aims to explore the feasibility and acceptability of using internet browsing history data for health research.

**Methods:**

Participants were purposively sampled to ensure representation from groups at risk of digital and health inequalities via community organizations and charities. We conducted semistructured and think-aloud interviews allowing participants to reflect on hypothetical research involving sharing their internet browsing data. The adapted theoretical framework of acceptability guided the interview structure and coding. The interviews were transcribed, coded in NVivo, and thematically analyzed. Patient and public involvement informed the study approach, participant-facing documents, and the interpretation of the findings.

**Results:**

Twenty participants (10 with a history of cancer and 10 without) were included in the study representing a range of age, gender, and ethnic and socioeconomic groups. Key themes focused on factors necessary for acceptability, including trust, transparency, and control and on perceived feasibility and individual willingness. Trust and transparency were fundamental to participants’ willingness to share data. Trust in researchers would have to be earned through clear communication, ethical data handling, and familiarity with a named research team. Privacy concerns were prominent, with participants wanting control over what was shared, particularly regarding nonhealth-related information (such as details related to banking) or activity related to others (such as their children). Potential use or misuse of data beyond the original research purpose caused more concern than the nature of the shared data itself. Digital literacy varied; many expressed concerns over the technical aspects of sharing data. Participants also doubted the value of their individual internet browsing history, for example, as they chose not to search for health information due to the prevalence of misinformation. However, they described wider benefits arising from internet browsing history research, such as potential advancements in early detection and opportunities to promote credible online sources.

**Conclusions:**

Participant recommendations balanced privacy concerns against the potential of internet history data for early diagnosis and health research. The study highlights ethical and inclusive approaches to health research using internet browsing history. Future researchers should consider defining the scope of health-specific data filters, providing user-friendly information and guidance for study participants, and ensuring that participants are able to contact research team members to build trust and facilitate data sharing.

## Introduction

For many medical conditions, good prognosis and effective treatment rely on timely diagnosis [1,2]. There is also a large economic burden of delayed diagnosis when disease becomes advanced or requires more invasive treatment, longer hospital stays, and greater impact on quality of life [3,4]. In particular, early detection of cancer is a national priority in England, with the National Health Service (NHS) Long-Term Plan aiming to diagnose 75% of cancers at an early stage by 2028 [5-7]. A patient’s pathway to diagnosis of cancer may begin weeks or months before they seek help from a health care provider, when they first notice changes in their body and make decisions about the next steps, including doing nothing, monitoring, self-managing (eg, through medication or rest), seeking external information, or consulting a health care provider [8]. Misinterpreting symptoms as not needing health care appears to be the most important reason for diagnostic delays, constituting approximately 60% of the overall time from first symptoms to diagnosis in cancer [9].

There is growing interest in exploring how the internet is used in response to symptoms and the decision to seek help. Accessing health information online prior to appointments can empower patients and enable more informed conversations with health care providers [10]. Individuals typically search for specific symptoms rather than diagnoses before visiting a health care professional [11,12], although over 40% of individuals opt against seeing a doctor after researching their symptoms [10,13,14]. Recent research has suggested that internet search activity history may provide valuable early digital signals of disease at an individual level. For example, there has been pilot work into the predictive utility of internet searches in relation to gynecological malignancies [15]. The researchers found differences in online search data between malignant and benign gynecological conditions, up to a year before general practitioner referral. Similar findings have been reported in lung cancer [16] and pancreatic adenocarcinoma [17]. However, studies have found variable opinions on sharing internet browsing history data [18-20], and there is a lack of diversity in studied populations. This has the potential to compound existing inequalities [21]. It is known that older adults, those from minority ethnic groups, and those with the lowest levels of socioeconomic status have differences in linguistics, disease rates, digital literacy, and help-seeking behaviors [21-23]; are less likely to use the internet for health information [24-26]; face barriers to internet access [22,27]; and are more likely to experience inequalities in cancer care and outcomes [28]. Disadvantaged groups are also less likely to be willing to share health information more generally [29,30].

Crucial to successful research into internet browsing history is ensuring this research is acceptable to research participants and achieves representative participation. There is currently a lack of knowledge about public and patients’ perceptions of acceptability of using individual level internet search data for medical research or the criteria that would enhance engagement. This is important as low or biased engagement could limit the potential of these studies. In this study, we define acceptability as a multifaceted construct, which reflects how potential research participants consider the proposed research to be appropriate [31]. We apply the adapted theoretical framework of acceptability [32], which highlights 8 constructs relevant to establishing acceptability, to explore the feasibility and acceptability of sharing internet browsing history data for health research among individuals with and without a history of cancer, including those from disadvantaged groups.

## Methods

### Participants and Recruitment

Recruitment took place through in-person engagement and recruitment posters (with a study email and phone number) at community organizations, local networks, and charities using a purposive sampling strategy to ensure representation of groups at higher risk of poorer health and digital exclusion, such as individuals experiencing higher levels of deprivation, older adults, individuals with lower educational attainment, and ethnic minority groups. Participants were adults of any gender, had access to a portable web-browsing–enabled device; were able to read and speak in English; and self-reported either no history of cancer or a history of cancer as an adult (excluding nonmelanoma skin cancer) to explore whether previous experience of cancer influenced willingness to share (see Multimedia Appendix 1 and Checklist 1 for full recruitment details). Individuals interested in taking part were screened in person or by phone for eligibility and sampling by MR, provided with study materials, and a date for the interview booked in pending consent. Interviews were conducted by MR or SA and took place between September 2024 and January 2025 at the university, community organization (London), or online (any location, England), whichever was preferred by the participant. Recruitment was ceased when we had captured a broad range of experiences and attitudes across a diverse group and achieved sufficient information power [33].

### Study Design

This research was led by an experienced health services research team, supported by a research assistant and a medical student. The team has expertise in psychology, anthropology, digital health, inequalities, clinical practice, and policy and was supported by patient and public involvement (PPI) contributors from diverse backgrounds (see the *Patient and Public Involvement* section), allowing us to bring together different perspectives during the study design, data collection, and analysis.

Taking a critical realist position, a mixed “reactive” and “reflective” “think-aloud” interview approach was used [34,35], in which participants were invited to speak their thoughts aloud in response to material prompted by the interviewers. After a “warm-up” think-aloud activity, participants were asked to think aloud while reading sections of a participant information sheet for a hypothetical future study, which asked people to share internet browsing data for health research (either prospective or retrospective data sharing). A second think-aloud activity involved participants viewing information on “Google Takeout” (a Google service that allows users to export data from other Google products, which has been used in previous studies to allow participants to share their internet history). To visualize this, participants were also shown an example image of internet search history and encouraged to view their own internet search history via their personal devices, if available. Participants were then asked broad questions on their attitudes to data sharing and the feasibility of taking part in internet sharing research projects. Interviews lasted approximately 60 minutes and were conducted by MR or SA.

### Data Analysis

Interviews were audio-recorded and professionally transcribed verbatim, and notes were made by the interviewer as a memory aid during the think-aloud portion of the interview. A framework analysis approach was employed, guided by the adapted theoretical framework of acceptability [32]. Familiarization and initial inductive and deductive coding were conducted by NCG using NVivo (version 1.6; Lumivero). To ensure consistency in the coding and thematic framework development, MR reviewed a subset of transcripts. Code definitions, grouping, and theme development were led by NCG and refined through discussion with MR, SS, and CD via a series of consensus meetings and validated with patient and public contributors. These discussions were informed by field notes kept by SA and MR, and analysis notes were kept by NG. Microsoft Excel was used to chart codes, develop a framework matrix, and compare responses between participants with and without a history of cancer.

### Ethical Considerations

Ethical approval was granted from Queen Mary University of London (QMUL) Research Ethics Committee (reference QME24.0548). The study protocol is available on OSF [36], and there were no deviations from this protocol. Informed consent was obtained from participants through a written consent form that was explained by the researcher following a detailed participant information sheet. Participation was voluntary, and participants could withdraw at any point of the study. Participants’ identifying information has not been included in this study. Participants were reimbursed £25 (US $33.50) for their time.

### Patient and Public Involvement

Three patient and public involvement contributors were recruited from the Centre for Cancer Screening, Prevention and Early Diagnosis (Queen Mary University of London) PPI pool. This is a working database of over 150 people who have expressed an interest in contributing to research. We requested engagement particularly from people aged 55 years or older and from a Black, Asian, or other minority ethnic background. PPI contributors discussed the overall study approach; reviewed the study documents, such as participant information sheets and interview guides; and validated the interpretation of the results and the study recommendations. Recommended changes that were implemented included improved wording on the study recruitment posters and clarification of the study purpose in the participant information sheets. PPI reporting is included in Checklist 2.

## Results

### Participant Characteristics

In total, 22 interviews were conducted. Two transcripts were excluded from the final analysis following team discussion. In both interviews, there was little engagement, the participants repeated information included within the question and were unable to provide details about their own experiences. As a result of this, the relevance and reliability of the data were insufficient for meaningful analysis. Half (n=10, 50%) of the participants had no history of cancer and half (n=10, 50%) had a history of cancer, representing a range of age, gender, and ethnic groups. Half of those with no history of cancer (n=5) and one third of those with a history of cancer (n=3) lived in areas described as high deprivation (ie, local postcode scores 1 or 2 according to the English Indices of Deprivation), where 1 represents the highest level of deprivation and 10 represents the lowest level of deprivation [37]. The full details of participant demographics are included in Table 1.

**Table 1.** Demographics of interview participants.

Demographic characteristic	Those with history of cancer (n=10), n	Those without history of cancer (n=10), n
Gender		
Male	2	5
Female	8	5
Ethnicity		
White/White British/White—Other	7	5
Black British/Black Caribbean	1	2
Asian British or Asian Other	2	3
Age range (y)		
19‐34	0	1
35‐44	1	2
45‐54	1	2
55‐64	6	2
65‐74	0	1
75‐84	2	2
Indices of deprivation		
1‐2 (highest deprivation)	3	5
3‐4	2	4
5‐6	3	1
7‐8	1	0
9‐10 (lowest deprivation)	1	0
Highest level of education		
Primary	0	0
Secondary	3	5
Tertiary (including professional qualifications)	6	3
No qualifications	1	1
Not provided	0	1

### Interview Results

The key themes focused on factors necessary for acceptability, including trust, transparency, personal control, and setting digital boundaries and on the perceived feasibility and individual willingness of participants to share internet browsing history for health research. Finally, participants offered recommendations on how future studies using this approach could facilitate trust and encourage participation.

#### Trust and Transparency as Foundations for Acceptability

Trust and transparency emerged as central factors influencing participants’ willingness to share their browsing history data. Trust was not automatic but needed to be actively earned through clear communication, familiarity with the research team, transparency about who would access the data, and integrity in data handling. Direct interaction with researchers, a clear understanding of the study’s purpose, and reassurance about data access enhanced participants’ sense of security and credibility.

*I’d feel comfortable because I’ve met you guys and you’ve explained clearly what your research is about, if it was a third party, then that’s kind of weird because I’ve never met that person.*

[P02, 35-39 years, female, no history of cancer]

Participants consistently articulated that their trust in the research team was closely tied to the perceived transparency of the team’s actions and intentions. Providing clear information about the researchers’ identities, data use, and opportunities to ask questions would foster reassurance and informed participation. Transparency also entailed setting clear boundaries around what data would be accessed and outlining the safeguards in place to protect privacy. Participants were generally willing to share information provided researchers strictly adhered to stated objectives, maintained ethical standards, and implemented robust data handling protocols, such as anonymization and secure deletion.

*It is always reassuring to see that you are following all the right things and that you delete information once you’ve collected it for your analysis.*

[P43, 75-79 years, female, history of cancer]

*Because of the sensitivity of that nature, I need to be 100% sure that this data are being used in the right way, are stored, accessed by a limited number of people for the purpose that is being clearly described and given consent for.*

[P54, 35-39 years, male, history of cancer]

Importantly, participants noted that it was not the nature of their data that caused concern but rather the apprehension that would arise if researchers deviated from the agreed scope of use. Such actions would raise doubts about the researchers’ intentions and significantly undermine trust. Thus, trust and transparency were seen as mutually reinforcing, whereby transparency was essential to the development and maintenance of trust throughout the research process.

*Providing that the researchers are open about what they’re doing and only do what they say they’re going to do, then that’s fine. If they start going beyond that then it would concern me … because it makes you question why they’re wanting to look for other things.*

[P47, 60-64 years, female, history of cancer]

*As long as it’s for research … If they are properly trained researchers, they’ll be ethical and respectful and will observe people’s confidentiality and privacy.*

[P43, 75-79 years, female, history of cancer]

Several participants highlighted the importance of having choice and autonomy, including the ability to opt out or withdraw consent at any time. These features reassured participants that their involvement remained voluntary and under their control.

*It’s quite good that it’s got an opt-out clause … you don’t have to take part if you don’t want to and that it won’t affect you in any way.*

[P26, 55-59 years, female, history of cancer, responding to the PIS]

Trust in the organizations managing search data was a fundamental factor in acceptability, with participants clearly differentiating between trusted public bodies and commercial companies. Institutional affiliations with reputable entities, such as the NHS (United Kingdom) and universities, were seen as markers of legitimacy, with logos, branding, and clear consent processes being mentioned as ways of reinforcing this trust. Commercial companies were often not trusted due to their profit-driven motives and concerns about data misuse, such as selling personal information to health insurance companies leading to potential discrimination.

*I’m not sure I saw a university or NHS trust logo … Just to give me that added confidence.*

[P43, 75-79 years, female, history of cancer, responding to the PIS]

*I think with the NHS it’s okay, but if it’s a private corporation or some commercial that engages with marketing stuff, then I’d be quite apprehensive about that.*

[P02, 35-39 years, female, no history of cancer]

*If it is for other reasons like health insurance, then they can target you and make you pay a higher premium, then I don’t think that is fair. And then I’m not happy with it.*

[P17, 60-64 years, female, no history of cancer]

#### Personal Control, Privacy Concerns, and Setting Digital Boundaries

Privacy and data security emerged as critical concerns for participants, reflecting broader mistrust in digital systems. The foundations of trust in data management were heavily dependent on participants’ perceptions of how their personal information would be treated. While some participants were open to sharing their data for medical research, many expressed reservations about the potential misuse of their personal information or information of other individuals using their devices, as well as data breaches. They highlighted the importance of maintaining control over their personal data.

*The only thing I’d worry is if certain things have been hacked.*

[P09, 20-24 years, male, no history of cancer]

*It’s that right to say yes or no, I have the right to keep my private things private.*

[P49, 60-64 years, female, history of cancer]

The concept of privacy was also viewed as inherently linked to transparency, with many participants seeking clear assurances about how their data would be used. Individuals described their internet history as deeply personal, a window into their habits, interests, and private moments and therefore a reflection of their day-to-day lives. Participants expressed strong concerns about the intrusiveness of using this type of data, emphasizing that it could feel like a violation of privacy if there was a risk of others accessing their data, making its use in research a potentially sensitive issue without clear ethical safeguards in place.

*I need to be convinced about this and get some reassurances that privacy issues will be dealt with accordingly. Otherwise, I find it intrusive*.

[P54, 35-39 years, male, history of cancer]

Interestingly, 6 out of the 20 participants, all those without a history of cancer, reported using incognito mode for reasons such as accessing restricted websites, watching YouTube, avoiding cookies or pop-ups, and conducting private searches related to health or banking.

Some participants expressed a strong desire to maintain autonomy over their personal information. While many would feel comfortable sharing health-related information, they were clear that they did not want to disclose any other personal or sensitive details, frequently citing financial information, political views, or family matters as examples.

*I like to have this agency over my digital data … I wouldn’t like such sensitive data to sneak in my internet use history and shared with irrelevant people.*

[P54, 35-39 years, male, history of cancer]

To overcome this, several participants emphasized the importance of having a filtering system that would allow them to share only health-related data, reassuring them that irrelevant or sensitive information, such as bank details or private communications, would not be accessed. This idea of a “health filter” was repeatedly mentioned as something that would make people feel more comfortable and in control of what was being shared.

*I do personally think that if this research is medical, then there has to be a way that all you are taking are medical notes.*

[P49, 60-64 years, female, history of cancer]

*I mean obviously no computer programme is 100% going to sift out everything that’s medical, but I think it would probably come close to answering my concerns.*

[P26, 55-59 years, female, history of cancer]

Participants did not state a preference for prospective or retrospective data collection. When prompted to reflect on whether knowing they would share their data with researchers would change their behavior, only 1 participant felt that they would be on “good behavior” and potentially delete personal activities. Other participants stated they were not concerned as they “had nothing to hide,” felt that “everything’s big brother as it is,” or that they would “forget.”

*I don’t believe people would necessarily be mindful about what they look, what they search, knowing that somebody else is checking on it.*

[P17, 60-64 years, female, no history of cancer]

#### Perceived Feasibility and Willingness to Participate in Health Research

##### Burden of Effort and Digital Literacy

Willingness to share internet browsing history was influenced by perceptions of effort and confidence with digital tools. While many participants were open to the idea, concerns emerged about the time and technical knowledge required to download and share these data. The process was seen by some as potentially tedious or confusing, particularly over a year-long period. One participant joked, “you’d be there all day with mine” [P40, F, 60‐64, history of cancer], reflecting anxieties about data volume and complexity.

Digital literacy varied considerably. While some participants felt comfortable navigating online platforms, others described themselves as “not expert” or relied on others for support. Tools such as Google Takeout were unfamiliar to many, and concerns were raised about storage, filtering content, and whether the process could be made simple and secure. For those less confident, the technical aspects posed as a potential barrier to engagement. These concerns were more commonly expressed by older participants, particularly those aged over 70 years, often unfamiliar with basic terms such as “browser” and relying on others for support; for example, 1 person told us: “I say I’m too old to learn*”* [P19, 75‐79, F, no history of cancer]. However, confidence did not always coincide with age, as some younger participants also expressed uncertainty.

*It’s too new to me, I didn’t know about Google Takeout and I didn’t know there was such a thing as a Google account.*

[P01, 70-74 years, male, no history of cancer]

*I don’t mind sharing it as long as it’s easy for me to actually share it.*

[P48, 55-59 years, female, history of cancer]

##### Personal Experience and Online Health Information

Participants with a history of cancer expressed notably higher levels of privacy concern, emphasizing the sensitive nature of their health data and the need for strict anonymity, whereas participants without a history of cancer, while still valuing privacy, were generally more pragmatic and less emotionally driven in their concerns. These differences suggest that a cancer diagnosis may intensify apprehensions about data sharing and potential identification. Some participants with cancer also highlighted that their main interaction with online health information came after their diagnosis, limiting the utility of their browsing history for early diagnosis (*“*… for me, … I don’t go looking for stuff on-on the internet until it’s happened”).

##### Innovation and Societal Impact

Participants recognized the potential for using internet browsing history data to improve individual health outcomes and also contribute to broader societal benefits. During the think-aloud interviews, many participants spoke more broadly about health, cancer, and internet search behavior, with some focusing on how people search for symptoms before diagnosis, while others mentioned improvements in accessing trustworthy information. Many commented on the culture of online health information, expressing frustration over “fake news” and the negative tone of many search results. There was hope that the research could help promote credible sources and bring trustworthy sources such as NHS websites to the forefront.

*I think it’s a really interesting premise, looking at if people are Googling their symptoms before they seek medical help … because intervening earlier might lead to earlier diagnosis and this would be very positive*.

[P26, 55-59 years, female, history of cancer]

*Google search symptoms can be a little bit doom and gloom … it would be great if people actually get more access [to] information … that [is] practical.*

[P02, 35-39 years, female, no history of cancer]

Participants expressed strong support for the use of digital tools and online behaviors in health contexts, particularly if related to improving early diagnosis and accessing trustworthy advice.

*If people can be helped earlier and better, then yes, I support it.*

[P17, 60-64 years, female, no history of cancer]

*Technology can really help people with health stuff.*

[P02, 35-39 years, female, no history of cancer]

*People like to be involved and want to know what effect it might lead to.*

[P43, 75-79 years, female, history of cancer]

*Only by sharing our data can progress be made in medical research.*

[P47, 60-64 years, female, history of cancer]

Early diagnosis emerged as a powerful motivator for data sharing and health research participation, with several participants emphasizing the importance of online data to prompt earlier help-seeking and therefore improve health outcomes. Participants’ experiences of cancer shaped their perspectives on the potential value of the research.

*I always feel really happy if I can help with a study that is going to absolutely make someone move forward with an early diagnosis.*

[P49, 60-64 years, female, history of cancer]

*We shouldn’t be going to stage three in cancer. I think we should be able to fix it at stage one.*

[P46, 45-49 years, female, history of cancer]

However, while people felt the research was worthwhile for early diagnosis, some questioned the usefulness of their own browsing history, describing it as “low value” or highlighting that they did not frequently use the internet to look up health questions.

## Discussion

### Principal Findings

This study explored the acceptability of using individual-level internet browsing history data for health research focusing on earlier cancer diagnosis. To our knowledge, no previous study has assessed the acceptability of using such data with respect to health inequalities and cancer.

In this study, participants placed greater confidence in public institutions such as the NHS and universities rather than commercial companies with fear of data misuse, discrimination, and profit motives being predominant reasons, similar to other studies [29,38,39]. Transparency played a foundational role in fostering trust, and this could be built and sustained through clearly communicating study aims, respecting participant boundaries, and keeping participants informed throughout the process.

Participants expressed ongoing concerns about the potential for their data to be hacked or misused once shared, reflecting broader mistrust in digital systems and the sensitive and private nature of internet browsing data. This sentiment was particularly strong among those with a history of cancer who linked their online searches to periods of emotional vulnerability and distress, particularly around diagnosis and treatment phases, similar to previous studies [40,41]. Younger participants took steps toward digital privacy, using “incognito mode” or alternative browsers or search providers seen as more secure, presenting a potential barrier for future research which relies on search history data.

To overcome these concerns of data intrusiveness, a key recommendation across interviews was the implementation of a robust filtering mechanism to isolate health-related data from other forms of personal information. Not only did this offer reassurance that sensitive or irrelevant information would not be accessed, but it also served as a practical mechanism to enhance participation by directly addressing concerns around data intrusion and potential misuse. Previous studies have applied health filters using a list of symptoms, diseases, and medications and their synonyms [15,42]. However, patient descriptions of symptoms may vary according to sociodemographic, linguistic, cultural, regional, and other factors [43,44], which may not be taken into account in lists developed from clinical lexicons. The definition of health-relevant information may also vary according to the condition a study focuses on: for example, negatively valenced web pages in individuals with depression [45], or typing speed and error rate in the presence of cognitive changes, such as in multiple sclerosis [46]. Future research approaches using browsing data will need to clearly define and communicate the limitations of any such filtering to participants.

Additionally, the study revealed wide variation in digital literacy, with both older adults and some younger participants expressing confusion around basic digital terms and concerns about making mistakes during the data-sharing process. This uncertainty about the technical aspects of using unfamiliar tools to download internet browsing data (such as Google Takeout) posed as a barrier, especially for those with limited digital confidence, highlighting the need for simple, user-friendly processes and tailored, individual support. Table 2 outlines key recommendations to enhance participation for future studies.

**Table 2.** Recommendations for research involving individual-level internet browsing history, as suggested by participants, linked to the study’s themes and the adapted theoretical framework of acceptability (ATFA) [32].

Area (ATFA construct)	Recommendations for research involving individual-level internet browsing history
Trust and transparency (ethicality, intervention coherence)	Provide clear and accessible information about the study and data use Use NHS^a^ or university branding to build trust where possible Ensure participants have control, including opt-out options Maintain open communication with participants Ensure each participant has a point of contact to direct queries Include clear information on the research team members including name and credentials
Privacy, data control, and digital boundaries (ethicality, trust)	Implement a robust filtering system to isolate health-related data and exclude nonhealth-related content (eg, finances, politics, schools); potentially including the option to remove browsing activity related to, or on behalf of, others such as friends and family Reassurance regarding anonymity, data storage, data protection, and confidentiality
Burden of effort and digital literacy (burden, self-efficacy)	Offer simple, step-by-step guidance on how to share search history Include visual aids or videos to support less digitally confident users Provide technical assistance when needed
Innovation and societal impact (affective attitude, perceived effectiveness)	Emphasize the societal benefits, such as improving early diagnosis Highlight how the research could help others Reassure participants that their contribution is meaningful and valued

a NHS: National Health Service (United Kingdom).

### Comparison With Prior Work

A recent review of harnessing internet search data as a potential tool for medical diagnosis identified ethical, bias, technical, and policy challenges [47]. In our study, several key factors emerged as influencing participants’ willingness to share their internet browsing data, consistent with previous research, which broadly categorized these into four key themes: (1) the relationship between the participants and the researchers, (2) fears and harms of data sharing, (3) purposes, and (4) benefits [48]. Similar recommendations were made in studies to share other digital data, such as loyalty card (purchase history) for academic research [49,50].

Across our study and other work, trust and transparency are integral components and prerequisites for willingness to share data. Trust is not a singular concept but multi-layered, operating at 3 distinct levels: interpersonal (trust in individual researchers), institutional (trust in health care or academic organizations), and systemic (trust in the digital infrastructure more broadly) [51]. Key “enablers” and “impediments” within these trust levels include sociodemographic factors and the reputation of the institution fitting into both categories [39]. Understanding these intersecting factors is key to ensuring research utilizing internet browsing history data for health research is equitable: for example, 1 study suggested that internet and social media data may be viewed by the public as more sensitive than electronic health record data [20]; another found that medical records were seen as the most sensitive type of data, but this was willingly shared with health researchers, while sharing banking data (seen as the second most sensitive) raised more concerns [50]. Attitudes to data protection and sharing may also vary by demographic group: for example, younger people and people with more education were more likely to choose an option to pay for a service to keep their data private, regardless of income level [52]. Additionally, people with long-term health conditions and those from ethnic minority backgrounds express higher concern about sharing data in general [53], and trust in researchers may vary by ethnic group [54]. Smart Data Research UK’s recent public engagement also found that while information about the purpose and benefits of the research positively influenced willingness for their data to be used in research, there was low trust in government and public institutions for these findings to impact policy [55]. This highlights the need to build trust at all levels to ensure that research using digital data targets those who would benefit the most from the earlier detection of cancer and to prevent the risk of exacerbating inequalities. Participants stressed the personal nature of establishing trust (“I’ve met you guys”), and prospective studies must consider the practical and resource implications of maintaining these relationships over the course of a study to support sustained participant engagement.

Our study also highlighted potential issues of feasibility related to sharing internet browsing history. Previous research has utilized “Google Takeout” as a method for data porting in this type of research. This study identified potential limitations to this approach: for example, participants used other browsers (eg, did not use Android devices); did not have a Google account or were not logged in. Further fragmentation of the digital ecosystem occurs through social media channels, in-app data access, and the impact of generative artificial intelligence on how people seek and review health information—such as only reading the in-browser artificial intelligence summary or asking ChatGPT [56]. A review of the use of internet search data in diagnosis research found no studies that integrated data from across different platforms and noted the complexity of standardizing such data for analysis and a need for supporting infrastructure and guidance on best practice.

Participants’ individual behavior also impacted feasibility; for example, they avoided “cyberchondria” by not using internet searches for health, used incognito mode to hide searches, and searched for health-related data for others (leading to potentially “messy” data). One study recruiting women with gynecological symptoms reported that one quarter of potential participants did not have a Google account and nearly 30% did not have relevant data or faced technical issues with sharing their data [15]. While the median age was similar in those included and excluded in the study, other potential demographic differences were not discussed. Other studies have identified similar issues, including participants consenting to take part in the study but deciding they did not want to share their Google searches [57]. One study that focused on internet use in older age groups (mean age 81 years) found that even among individuals who used computers, 22/76 (29%) did not conduct any searches during the duration of the study, and a further 12 did not conduct any searches in a particular 3-month period [58]. Some studies have only included individuals with a Google account [59-61], but this may impact the utility of this type of research approach in sociodemographically diverse populations.

This study found that in most domains, participants with and without a history of cancer shared similar perspectives. It is possible that the differences in the use of incognito mode between the participants with and without a history of cancer may relate to individual variability in digital literacy, as other participants were not aware of this function. This was not necessarily limited by age or education level, as those who used it were within age brackets from 20 to 64 years. In contrast, participants with a history of cancer expressed notably higher levels of privacy concern, although they were similarly willing to share their data given adequate safeguards. We speculate that this may be due to their closer experiences with the health system, potentially identifying the nature of rare cancers and their focus on their browsing activity postdiagnosis. Qualitative studies in cancer survivor populations have found similar willingness to share and made similar recommendations for building trust [62], and comparisons of individuals with and without a history of cancer found similar willingness to share information and a higher willingness in those with a history of cancer to share sensitive information (such as genetic information) [63]. This suggests an opportunity to examine in more detail how individual health experiences may impact willingness to share data for research related to specific health risks.

Studies that have involved sharing of internet search logs and health data with researchers have typically been retrospective, and there is limited evidence regarding differences in search patterns in study participants who donate data prospectively [47]. A study of the observer effect on social media use did find differences in posting behavior including frequency and topics [64]. Participants were prompted to discuss this during the interview and reported that it would not impact their (long-term) browsing activities. Nevertheless, this remains an important area to explore.

### Strengths and Limitations

This study aimed to explore the acceptability of sharing internet browsing history for research, ensuring representation from groups more at risk of experiencing digital and cancer inequalities. We know that 3% of the population of the United Kingdom do not use the internet, facing barriers such as cost, poor connectivity, and lack of digital skills and that people in this group are more likely to be older and/or earning less [65]. Any future research of this kind would require individuals to use the internet; this required us to find a balance when recruiting and designing the methods. Offering both online and face-to-face interviews (at a convenient location) improved accessibility and allowed those who only use the internet on their phone or on shared devices (eg, library computers) to participate. Additionally, we purposively sampled groups affected by health and digital inequalities, enhancing the relevance of the findings across diverse populations. The participants with a history of cancer were more likely to have a tertiary level of education and showed more variability in whether they lived in more or less deprived areas. However, as limited comparisons were made between the groups, we feel this sample shows sufficient variability across different groups affected by health and digital inequalities. The interviews were restricted to English to support the think-aloud exercises, and as current internet history research has focused on single-language searches. However, future approaches should consider how the internet activity of more diverse populations can be included, particularly as diaspora populations may choose to seek and act on health information from their country of origin [66]. While self-selection bias is possible as participants may have had a preexisting interest in health research or felt comfortable discussing digital data, the diversity and depth of responses suggest that a broad range of perspectives was captured. This was an exploratory study (n=20), and future studies may wish to explore specific barriers and solutions in populations at different risks of exclusion.

### Conclusion

This study highlights the steps required to improve the viability of internet browsing data to support earlier diagnosis and enhance public health outcomes. While concerns around privacy, data use, and institutional trust persist, they were not seen as insurmountable. Participants generally expressed positive attitudes toward this innovation, when use is clearly linked to tangible health benefits, provided there are ethical safeguards, simple guidance, and opportunities for consent. The integration of internet browsing data with a patient’s medical records may present an opportunity for identifying early signals of disease and holds promise for future research.

Importantly, this research approach offers unique opportunities to reach individuals who may face barriers to accessing traditional health care services, potentially reducing health inequalities and promoting more inclusive approaches to early diagnosis. However, the implementation of this approach requires careful attention to issues of accessibility, digital literacy, and infrastructure; otherwise, the same approach is at risk of inadvertently exacerbating existing sociodemographic disparities.

To fully harness the benefits of internet search data in health care, systems must become more integrated, secure, and person-centered. As global health pressures grow, such as the rising incidence of cancer, leveraging digital data responsibly offers a path toward more preventative and sustainable health care strategies.

## Supplementary material

10.2196/82009 Multimedia Appendix 1 Participant recruitment, interview guide, and sample participant information sheet.

10.2196/82009 Checklist 1 COREQ checklist.

10.2196/82009 Checklist 2 GRIPP checklist.

## Abbreviations

NHS: National Health Service
PPI: patient and public involvement
