# Supplementary material for: Acceptability of Sharing Internet Browsing History for Cancer Research: Think-Aloud and Interview Study
Source: JMIR Cancer. 2026 Feb 2;12:e82009. doi: 10.2196/82009 (PMC12863654; doi:10.2196/82009)
Supplement: Multimedia Appendix 1 [file cancer-v12-e82009-s001.docx]

# Multimedia Appendix 1

- Appendix A: Participant recruitment
- Appendix B: Interview guide and sample Participant Information Sheet

# Appendix A: Participant recruitment

Table S1: Inclusion and exclusion criteria

| **Inclusion Criteria** | **Exclusion Criteria** |
| --- | --- |
| Participants met the following criteria:   - Adults of any gender, aged 18 years or older. - Resided in the UK. - Had access to a portable device that supported web browsing (e.g., smartphone, tablet, or laptop). - Were capable of providing informed consent. - Were either:   - An individual with no history of cancer, or   - An individual with a history of cancer as an adult (excluding non-melanoma skin cancer). | Individuals were excluded from participation if they:   - Were unwilling or unable to provide informed consent. - Lacked access to an appropriate digital device. - Were unable to understand written and/or verbal English. - Were deemed vulnerable or lacked the capacity to give fully informed consent. |

Table S2: Sampling approach to ensure data representation

| **Participant Type** | - No history of cancer *or* - History of cancer as an adult, excluding non-melanoma skin cancer. |
| --- | --- |
| **Digital use** | - A range of operating systems (Android, iOS) and search engine preferences. |
| **Demographic Profile** | - **Deprivation:** Individuals residing in areas ranked within the most deprived IMD deciles (1 and 2) according to *Opendatacommunities* Postcode Lookup. - **Age:** Individuals aged 55 and above, as this group faced increased cancer risk and digital exclusion. - **Education:** Individuals whose highest level of education was compulsory education or less (ONS categories 0-2). - **Ethnic Minority Groups:** Representation was considered alongside other demographic factors to ensure inclusivity, particularly among groups disproportionately affected by health and digital inequalities. |

# Appendix B: Interview guide and sample Participant Information Sheet

Introduction

My name is [insert name] and I’m a researcher at Queen Mary University of London. I am not a healthcare professional, nor do I have any contact with your doctor.

I’m interested in how you feel about taking part in health research which looks at how people use the internet. You can stop and ask me if you don’t understand any of the terms we will use in this interview. Is there anything that you would like further information about before we begin?

We would like you to look at parts of a Participant Information Sheet that explains this type of study, and let us know any thoughts or questions that you have about it. We will also ask you to try to access your internet history – don’t worry we will not ask you to show it to us. Afterwards we will chat about your responses so I can check I have understood your views. There are no right or wrong answers, we want to understand your opinion.

Do you have any questions about the study?

You have signed the consent form. Are you happy for me to start recording?

Part A: Think Aloud – Participant Information Sheet

| **Part A Introduction** | In a moment, I will ask you to press start on the screen in front of you. It will take you through to a section of a Participant Information Sheet which explains a future study looking at how we can use information about how people use the internet to manage their health. A Participant Information Sheet explains to the person taking part in the study what the study is about, and what they can expect to happen. You will have read a Participant Information Sheet as part of agreeing to take part in this study.  There are 3 pages for you to click through, which each includes one part of a Participant Information Sheet. A real one would contain more sections than this.  On each page I will ask you to say everything you are thinking as you read the information. This could include questions that you might want to ask the researcher or any information that is not clear. I would like to emphasise this is not a test, and you can take your time reading. You don’t need to plan what you are saying so you can act as if I am not here. I will stay quiet and we can talk about your responses at the end. I may ask some questions if you are silent for a long time. You can say anything and everything you are thinking. |
| --- | --- |
| **Warm-up activity** | To begin with we will do a short warm-up activity. When you are ready, please press start and follow the instructions on the page.  *Participants will be asked to follow the test activity (see Appendix 2 for more details)*  *Prompts*   - *What are you thinking now?* - *Remember to keep speaking out loud what you feel about the information.*   *When you have finished the page, please press next to move onto the next page.* |
| **Part A Activity** | Ok, when you are ready, please press start.  *Participants will be asked to read one of two versions of the Participant Information Sheet (see Appendix 2 for more details)*   \| **Version** \| **Timeline** \| \| --- \| --- \| \| Version 1 \| Retrospective \| \| Version 2 \| Prospective \|   *The final page will invite participants to click a box to register their interest in taking part in future research.*  *Prompts*   - *What are you thinking now?* - *Remember to keep speaking out loud what you feel about the information.* - *When you have finished the page, please press next to move onto the next page.* |
| **Part A**  **Questions** |  |
| Clarification | - I noticed that you mentioned that you thought […] was… Can you tell me a bit more about that? - I noticed that you made a comment about […] could you elaborate on that? |
| Understanding the study | - Can you explain, in your own words, what the study is about? - What do you understand by ‘internet use history’? - How do you think taking part in a study like this would affect you? |

Part B: Browser history data

| **Part B Introduction** | Thank you for answering those questions. In this part of the study, we are going to introduce you to one way in which you could be asked to share your internet history data in a research study, and ask you to have a look at *your own* internet and/or smartphone history. You do not need to share anything in your internet history with us, we are interested in understanding how you find it. |
| --- | --- |
| **Part B Establish system familiar to participant** | - What type of device have you brought with you today? [*Interviewer to determine brand and operating system]*   - *Is this the device that you use most often?* - What type of browser do you use to look for information on the internet?   *Note for researcher in case asked, export is available on Google Chrome if signed in, Safari, and Edge. No export available in Firefox.* |
| **Part B – Responding to Takeout** | - On the next page is a short explanation about Google Takeout, which is one way that researchers might ask you to share your internet history. Have you used it before?   - Do you have any comments or questions?   On the next screen, is an example of what someone’s Takeout data would look like. Please take a few moments to look at it and describe what you see and understand. |
| **Part B Activity – any browser** | - When you are ready, I would like you to open your browser history, and tell me what you’re thinking as you find it.   - [Allow time to find it, guide if do not know how to find it] - Can you tell me about what you can see?   - *Did you know that your internet activity is stored in this way? How do you feel about it?*   - *Prompt about different types of searches, sites visited, etc.*   - *Would you be willing to share this data for health research?* |
| Clarification | - I noticed that you mentioned that you thought […] was… Can you tell me a bit more about that? - I noticed that you made a comment about […] could you elaborate on that? |

Part C: Semi-structured interview

| Takeout/browsing data feasibility [Burden] | Next I’m going to ask you some more questions about sharing your internet history.   - Do you access the internet on a shared device?   - *Can you tell me a little bit more about who uses it and what they use it for?*   - *Does this affect how you feel about sharing internet history?* - How did you find looking at your browser history? - Do you usually do anything to manage your internet history?   - *Do you ever delete any history?*   - *Do you ever use incognito windows?*   - *[If relevant] Have you managed the information that is collected about you in your Google account?* |
| --- | --- |
| Self-efficacy | - If you needed to download your data in the future, what type of guidance would you want to support you? |
| Attitudes towards data sharing (general) [Ethicality] | - Can you tell me how you would feel about sharing your browser history for research?   - *Are there any parts of your internet history that you would feel more or less comfortable sharing?*   - *If parts could be taken out before sharing, what would they be?* - How would you feel about sharing other types of information for research, such as your YouTube history or other information that was shown in the Takeout video?   - *Follow up on any comments in Think Aloud section.*   - *Are there any particular types of activity that you would not like to share?* |
| Trust  [Ethicality] | - Can you tell me about who you would feel comfortable viewing your internet history?   - *[Depending on answers to previous question] Are there any uses that you would not like to share your internet history? [e.g. commercial research]*   - *Is there anyone that you would not feel comfortable viewing your internet history?* |
| Opportunity costs | - Can you explain what worries you about sharing your internet history for research?   - *What, if anything, would make you feel more secure?* |
| Perceived effectiveness / Coherence | - Can you tell me what benefits you can see, if any, to sharing your internet history for research?   - *What information would help you make a decision about the benefits of this type of research?* - Do you feel that this type of research is worth doing? (Why or why not) |
| Additional questions specific to the PIS Version | PIS v1 (retrospective, EHR access)  PIS v2 (prospective, EHR access)  Retrospective   - In the participation sheet you looked at, the study asked for your past internet use. How would you feel about sharing your internet use for a period in the future?   - would knowing that your internet use is going to be looked at change how you use the internet?   - Is there any reason that you prefer [option]?   Prospective   - In the participation sheet you looked at, the study asked to share your future internet use. How would you feel about sharing your past internet use?   - Is there any reason that you prefer [option]?   EHR access   - In the participation sheet you looked at, the study asked to be able to link to your health records.   - Would you feel differently about taking part in a study that did not ask to link to your health records? |
| Flag for follow-up | Has this interview raised any new opportunities or concerns for you around how you can use the internet for health?  *Let them know will send links to information related to areas of concern or more learning, e.g. from AbilityNet.* |

**Wrapping up**

Is there anything else you would like to tell me?

Are you still happy for us to transcribe the recording of this discussion and use it as part of our research?

How have you found this discussion?

Do you have any questions?

You can now dispose of your questionnaire.

Thank you for your time.

Give sources of further support handout.

Provide participant with £25 retail voucher.

Example content of Participant Information Sheet (PIS)

Example PIS was presented on Qualtrics, allowing participants to navigate between information.

| **Version** | **Timeline** | **Electronic health record access** |
| --- | --- | --- |
| Version 1 | Retrospective | Requests access |
| Version 2 | Prospective | Requests access |

PIS Version 1

| Page# | Content |
| --- | --- |
| 0 | *Interviewer enters participant number and date, and moves onto the next page.* |
| 1 | *When the interviewer asks, please press START to complete the warm-up activity.* |
| 2 | *This is a warm-up activity to get you used to ‘thinking aloud’. This is not a test.*  *Please read the following section which is taken from the NHS website, and say everything you are thinking as you read the information.*  **How to be more active**  Regular physical activity is good for our bodies and minds, but it can be difficult to get started – especially if we're not particularly sporty or have not done any exercise for a while.  Here are some simple ideas to build more activity into your day, tips to stick with it and free exercise plans to help you get going.  ***Ways to increase activity in daily life***  Not all of us are naturally sporty and it can be hard to know where to begin.  Start small by finding easy ways to fit more activity into your daily life and build up from there. For example:   - stand rather than sit when you can, like on train or bus journeys, or try getting off the bus a stop or two early and walking the rest of the way - if you have to drive somewhere, park a little further away than you need to – even just the far end of the car park adds a little extra activity - take the stairs instead of the lift or walk up escalators when you get the chance |
| 3 | *When the interviewer asks, please press START to read the first section of the Participant Information Sheet* |
| 4 | **Invitation Paragraph**    We would like to invite you to participate in the EXPLORE Study. You should only participate if you want to; choosing not to take part will not disadvantage you in any way. Before you decide whether you want to take part, it is important for you to understand why the research is being done and what your participation will involve.  **What is the purpose of the EXPLORE study?**    The EXPLORE study aims to understand what we can learn about people’s health from their internet use history. In this part of the study we want to learn more about how people use the internet to manage their health before cancer was suspected or diagnosed by their doctor, and how this is different to people who have not had suspected or diagnosed cancer. This can help us improve how advice and care is offered to people in the future.  *When you have finished this page, please press NEXT to read the next section of the Participant Information Sheet* |
| 5 | **What happens to me if I take part?**  If you decide to take part, we will ask you to download your internet history from the past year and share it with us. We will provide detailed instructions, or a member of our team can help you do that.  We will also ask for your permission to access your electronic health records from the past year. These are records about the care you’ve received from the NHS, such as conditions you’ve been diagnosed with, medicines you’ve been prescribed, and results of tests.  *When you have finished this page, please press NEXT to read the next section of the Participant Information Sheet* |
| 6 | **What happens to my data?**  Your data will be kept securely and used only in the way described in this information sheet. Only researchers directly involved in the study will have access to data which identifies you.  We will use a computer programme to help us select your internet activity that could be related to health, and we will delete any other internet activity including anything that could identify you. Your name will be removed from your medical records and you will be given a unique identification number.  We will look at the internet activity data of people who take part in the study to understand the types of things people search for around their health or the way they search. We will compare people’s internet activity with their medical records to understand what people may search before they get help for an issue with their health.  *When you have finished this page, please press NEXT to read the next section of the Participant Information Sheet* |
| 7 | Would you be interested in taking part in this type of research in future? *Please tick the box if you would like to be contacted about this type of research.*  Thank you for reading this Participant Information Sheet. The interviewer will have some questions for you. When the interviewer asks, please press NEXT to move onto the next page. |
| 8 | **What is Google Takeout?**  Google Takeout is a service available to people with a Google account. It helps people download copies of their data from their Google account to save it in another location if they wish.  This could include:   - Chrome (internet history) - Documents - Calendar - Photos - YouTube videos   People can choose the types of Google services they download data from. For example, someone could choose to download only Google Chrome history and YouTube videos.  People might use Google Takeout for lots of different reasons. This could include saving their data, or moving it to another service. For example, moving their Photos from one account to another.  It is also possible to safely share this information with another person, such as a research study. |
| 9 | This is an example of the type of activity that might be recorded in your Google Account:  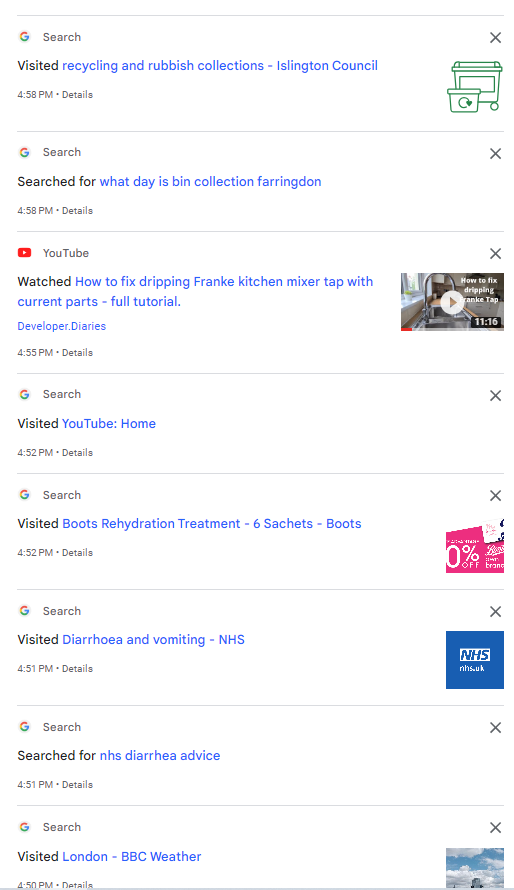 |
| 10 | Thank you for taking part in this study. Please press SUBMIT to close. |
