# Supplementary material for: Acceptability of Sharing Internet Browsing History for Cancer Research: Think-Aloud and Interview Study
Source: JMIR Cancer. 2026 Feb 2;12:e82009. doi: 10.2196/82009 (PMC12863654; doi:10.2196/82009)
Supplement: Checklist 2 [file cancer-v12-e82009-s003.docx]

# GRIPP2 Short Form

GRIPP2 short form

| **Section and topic** | **Item** | **Reported on page No** |
| --- | --- | --- |
| 1: Aim | Report the aim of PPI in the study | 4 |
| 2: Methods | Provide a clear description of the methods used for PPI in the study | 4 |
| 3: Study results | Outcomes—Report the results of PPI in the study, including both positive and negative outcomes | 4 |
| 4: Discussion and conclusions | Outcomes—Comment on the extent to which PPI influenced the study overall. Describe positive and negative effects | 4 |
| 5: Reflections/critical perspective | Comment critically on the study, reflecting on the things that went well and those that did not, so others can learn from this experience | 13 |
